# Evaluation of timed dexamethasone eye drops to prevent proliferative retinopathy of prematurity: a study protocol for a randomized intervention, multi-centre, double-blinded trial (DROPROP)

**DOI:** 10.1186/s12887-025-05673-x

**Published:** 2025-04-28

**Authors:** Ann Hellström, Mariya Petrishka-Lozenska, Aldina Pivodic, Anders K. Nilsson, Ulrika Sjöbom, Ingrid Hansen Pupp, David Ley, Lotta Gränse, Hanna Maria Öhnell, Gunnar Jakobsson, Karin Sävman, Lois E. H. Smith, Pia Lundgren, the DROPROP collaboration group, Magnus Domellöf, Kristina Teär Fahnehjelm, Uwe Ewald, Eva Albinsson, Erik Normann, Afsaneh Alibakhsh, Nikica Tomašić, Fredrik Ingemansson, Pierfrancesco Mirabelli, Despina Tsamadou, Jenny Wallander, Evangelos Tsigkoulis, Stefan Löfgren, Sofie Eriksson, Karin Hochhard Sandgren, Ylva Friberg-Riad, Liv Vallin

**Affiliations:** 1 The Sahlgrenska Centre for Pediatric Ophthalmology Research, Department of Clinical Neuroscience, Institute of Neuroscience and Physiology, Sahlgrenska Academy, University of Gothenburg, Gothenburg, Sweden; 2 Department of Ophthalmology, Sahlgrenska University Hospital, Region Västra Götaland, Gothenburg, Sweden; 3 Department of Clinical Sciences Lund, Pediatrics, Lund University, Skåne University Hospital, Lund, Sweden; 4 Department of Clinical Sciences Lund, Ophthalmology, Lund University, Skåne University Hospital, Lund, Sweden; 5 Department of Ophthalmology, Sahlgrenska University Hospital, Mölndal, Region Västra Götaland Sweden; 6 Department of Pediatrics, Institute of Clinical Sciences, Sahlgrenska Academy, University of Gothenburg, Gothenburg, Sweden; 7 Department of Neonatology, The Queen Silvia Children’s Hospital, Sahlgrenska University Hospital, Gothenburg, Region Västra Götaland Sweden; 8 The Department of Ophthalmology, Boston Children’s Hospital, Harvard Medical School, Boston, MA USA

**Keywords:** Retinopathy of prematurity, Dexamethasone, Preterm infants, Eye drops

## Abstract

**Background:**

As the survival rate of preterm infants continues to rise worldwide, more infants are at risk of developing sight-threatening retinopathy of prematurity (ROP). Destructive retinal laser treatment and intravitreal injections of anti-vascular endothelial growth factor (VEGF), factor, which have potential systemic side effects, are necessary to prevent blindness in severe cases of ROP. Off-label use in clinical settings suggests that dexamethasone eye drops, 1 mg/ml, may prevent the progression of ROP to severe disease (Type 1 ROP) requiring treatment. Our current study aims to assess the efficacy and safety of timely administered dexamethasone eye drops to reduce the need for laser or anti-VEGF ROP treatment in preterm infants.

**Methods:**

In a randomized prospective interventional, multi-centre, double-blinded trial, we plan to include 100 infants with severe ROP born before gestational age 30 weeks in Sweden. Infants will be randomized to intervention with dexamethasone eye drops (1 mg/ml) *(n* = 50) or placebo, saline (*n* = 50) until either ROP is resolved or severe ROP (Type 1 ROP) development occurs, fulfilling ROP treatment criteria. Eye drops will be administered one drop per day or every other day, depending on the severity of ROP, with a maximum duration of 12 weeks. The primary objective is to evaluate whether dexamethasone intervention reduces the proportion of infants developing Type 1 ROP compared to infants receiving a placebo. Adverse events and potential side effects will be recorded, such as high intraocular pressure and growth restriction. Levels of cortisol in saliva and glucose in urine will be measured repeatedly. Secondary outcomes will include the timing of ROP progression, the recurrence rate after ROP treatment and retinal morphology. An ophthalmological follow-up will be initiated at 2 and 5.5 years of age, evaluating visual acuity, refractive errors, strabismus, retinal morphology and ophthalmological complications. All outcomes in the study will be compared between the infants receiving dexamethasone intervention and those receiving placebo.

**Discussion:**

Timely administration of dexamethasone eye drops may prevent severe ROP from progressing to Type 1 ROP, which requires treatment. This study aims to assess the efficacy and safety of dexamethasone intervention to support its clinical use and national guidelines.

**Trial registration:**

EudraCT, 2020–004933-19, registered in January 2021 and CTIS, 2023–505318-97–00, registered in August 2023.

**Clinical trial number:**

Not applicable.

**Supplementary Information:**

The online version contains supplementary material available at 10.1186/s12887-025-05673-x.

## Background

Advancements in neonatal care have improved survival rates of preterm infants worldwide, leading to a growing number of infants suffering preterm-related morbidities [1–3]. Retinopathy of prematurity (ROP) is one of the leading causes of childhood blindness worldwide and primarily affects extremely preterm infants [4, 5].

In the initial phase of ROP, which occurs shortly after birth, blood vessel development in the retina is halted. During the next several weeks to months, the peripheral neural retina develops slowly and remains avascular. Increasing ischemia and inflammation in the avascular retina can lead to pathological neovascularization driven mainly by vascular endothelial growth factor (VEGF). In the worst-case scenarios, this neovascularisation leads to retinal detachment and blindness [6–8]. About 5–10% of ROP cases are severe enough to require treatment with retinal laser photocoagulation and/or with intravitreal anti-VEGF injections [4]. International guidelines regulate treatment indications based on ROP classification [9]. ROP is classified into ROP severity—stages (0 to 5) and retinal localization—zones (I to III) [10]. The presence of plus disease, tortuosity and dilation of central retinal vessels is a crucial sign for deciding the need for treatment. Severe ROP with indications for treatment (Type 1 ROP) is defined as any stage of ROP in zone I, with plus disease; ROP stage 3 in zone I, without plus disease; ROP stage 2 or 3 in zone II, with plus disease. Severe ROP requiring frequent follow-up but not fulfilling treatment criteria (Type 2 ROP) is defined as ROP stage 1 or 2 in zone I, without plus disease and ROP stage 3 in zone II, without plus disease [9]. Laser photocoagulation, which destroys the peripheral avascular retina, releasing angiogenic factors, helps reverse pathological vaso-proliferation. Laser treatment is often performed under general anaesthesia, which can be challenging for these vulnerable infants. Potential side effects after laser treatment are retinal haemorrhage, visual field loss, myopia, and retinal detachment [11]. Intravitreal injection with anti-VEGF can be performed under sedation, thereby avoiding the risks associated with general anaesthesia. However, intravitreal anti-VEGF agents enter the bloodstream, suppressing VEGF over an extensive period (ranging from weeks to months) with potential long-term systemic effects [12].

Dexamethasone is a synthetic glucocorticoid with anti-inflammatory effects. It is widely used in adults to treat ocular surface and intraocular inflammation, as well as to manage complications from branch retinal vein occlusion and diabetic retinopathy [13, 14].

Research in both animals and infants has shown the involvement of inflammatory mediators in the development of ROP [15–19]. Dexamethasone eye drops are commonly used in preterm infants to reduce local inflammation after laser treatment for severe ROP. Öhnell et al., in a retrospective uncontrolled study, reported regression of severe ROP in infants following dexamethasone eye drops prior to planned laser treatment [20], and Yagi et al. observed comparable results in a pilot study [19]. If treatment with dexamethasone eye drops efficiently prevents the progression of ROP with less severe disease and fewer cases requiring treatment, infants may avoid general anaesthesia and retinal sequelae. However, no clinical trial has yet evaluated the efficacy and safety of administering dexamethasone eye drops to preterm infants with severe ROP. Topical administration of dexamethasone has been studied in a mouse model of ROP, oxygen-induced retinopathy (OIR), and when administered concurrently with oxygen exposure in OIR, a protective effect has been reported [17]. However, the timing of administration appears to be critical, as topical dexamethasone administered before the peak of neovascularization in OIR suppressed neovascularization by 30%, whereas later dexamethasone treatment had no effect [18]. In OIR, optimally timed topical dexamethasone administration and reduced neovascularization were associated with enhanced retinal mitochondrial function and decreased inflammatory response in immune cells [19]. Thus, research from OIR supports the clinically reported findings that topical dexamethasone may be an effective strategy to prevent severe ROP. Understanding the importance of timing in steroid treatment may explain previous conflicting results regarding intravenous and oral corticosteroids and ROP development [21, 22].

Studies on the efficacy and safety of dexamethasone eye drops to prevent severe ROP needing treatment in preterm infants are warranted. We designed a prospective, randomized, double-blinded, controlled, multi-centre intervention trial to include preterm infants with severe ROP not fulfilling treatment criteria, i.e. infants with Type 2 ROP and infants with stage 2 ROP in posterior zone II. We aim to reduce the number of infants with severe ROP needing treatment by 60% with the dexamethasone intervention compared to placebo.

## Methods/design

### Study design

**Fig. 1:**
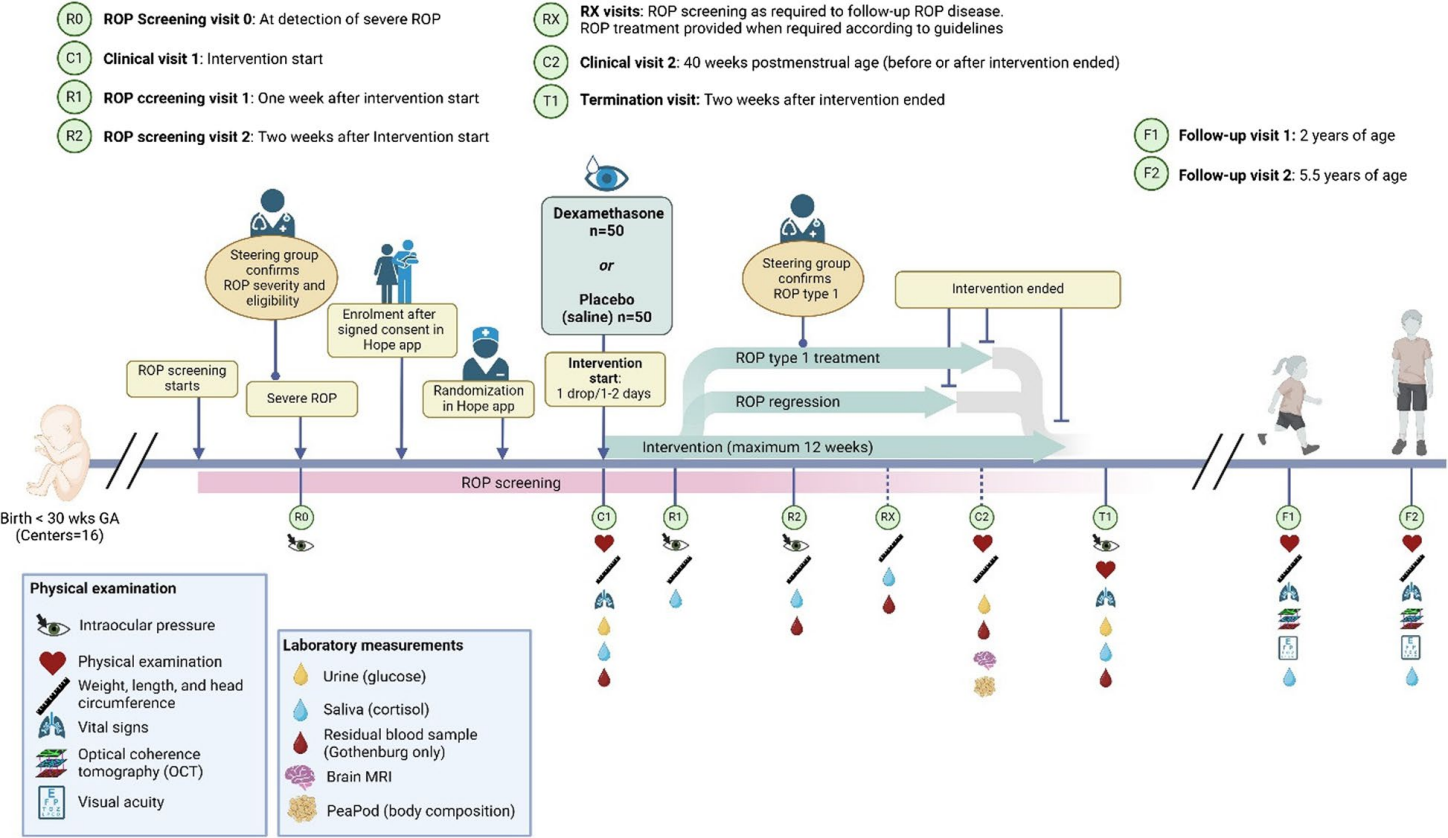
Schematic overview of the complete study schedule

The study is a national multi-centre, randomized, double-blinded, controlled prospective trial. The intervention group receives dexamethasone eye drops, 1 mg/ml (Dexafree®), while the control group receives placebo, saline (Drop-it®). The study consists of an intervention phase (main) study and an extension phase (follow-up) study. Figure 1 gives a schematic overview of the complete study schedule.

Professor Ann Hellström (ann.hellstrom@medfak.gu.se) is the primary sponsor and principal investigator (PI) in this study. The steering group is composed of three experienced paediatric ophthalmologists. The first infant was enrolled on 2022/09/22, and recruitment is ongoing.

### Study population

The following centres in Sweden participated in the study: Sahlgrenska University Hospital (Gothenburg), Karolinska University Hospital (Stockholm), the university hospitals in Umeå, Uppsala, Örebro and Linköping. Södra Älvsborgs hospital, Skaraborgs hospital (Skövde), Ryhov hospital (Jönköping), and the hospitals in Karlstad, Växjö, Gävle, Östersund, Västerås and Eskilstuna.

### Inclusion and exclusion criteria

Infants are eligible if they fulfil the following criteria:

- Infants born with a gestational age (GA) < 30 weeks.
- ROP stage 1 or stage 2 without plus disease in zone I.
- ROP stage 2 or stage 3 in posterior zone II without plus disease, with or without notch in zone I.
- ROP severity must be documented using wide-field digital imaging system photography (RetCam), and eligibility must be confirmed by at least two of three experienced paediatric ophthalmologists in the steering group.
- Signed informed consent by the parents/guardians (oral and written information).

The exclusion criteria are:

- Ongoing ocular infection.
- If the neonatologist or ophthalmologist considers the infant unsuitable for the study.

### Eligibility, randomization and blinding

Infants evaluated for eligibility for enrollment in the study will be identified at routine ROP screening by the local ophthalmologist. ROP evaluations will adhere to clinical screening practice according to national guidelines. RetCam will be used at the ROP screening to document the extent of ROP disease, and images will be saved in the clinical record file. When the ROP screening ophthalmologist evaluates eligibility, the PI and the steering group will be contacted. Images will be evaluated, and eligibility for inclusion must be confirmed by at least two of three ophthalmologists in the steering group.

Parents/guardians will be given oral and written information about the study via the HOPE Parent Application (Addi Medical) and asked to participate if the infant is eligible. The signed informed consent will be collected before any study-specific data is reported and the infant is randomized. Randomization will be performed stratified by centre by a study nurse using an electronic application (HOPE). After randomization, the study nurse will provide eye drops to the assigned neonatal nurse in the neonatal intensive care unit (NICU) for administration or to the parents or guardians if the infant is discharged from the hospital. In case of problems regarding the study eye drops, a randomization list is safely stored in a locker at the NICU and available 24 h/day in case of emergency and safety issues. All investigators, ophthalmologists, staff, and patients/guardians, except pharmacists, are blind to the participants’ eye drop assignments. The single-dose containers for dexamethasone and placebo are similar, and identification of drop content will not be visible to an untrained eye.

### Intervention

Study patients will be randomized to receive eyedrops with either dexamethasone 1 mg/ml (Dexafree®) in single-dose containers supplied by Thea Nordic or placebo, saline (Drop-it®) also in single-dose containers. Saline (Drop-it®) is a sterile, buffered saline without preservatives, with the same sodium chloride content as the tear film. The dose of dexamethasone has been adjusted to maintain efficacy while minimizing the medication dosage.

Based on the severity of ROP, the eye drops are administered at a dosage of one drop every other day to one drop daily in affected eyes, Table 1. The maximum intervention duration is 12 weeks. The dosage will be adjusted within the protocol’s range if ROP regresses or progresses. When discontinuing intervention, drops are tapered over one week; if the infant receives one drop daily, the dosage should be reduced to one drop every other day for one week before discontinuing intervention. After laser treatment, the infant will receive dexamethasone 1 mg/ml to reduce post-laser inflammation; the dose and duration will be determined by the ophthalmologist in charge, based on local routines. If anti-VEGF treatment is planned, all eye drops will be stopped immediately to reduce the risk of infection and endophthalmitis in cases of dexamethasone intervention. To reduce systemic absorption of the medication, we inform caregivers to tilt the baby's head to the left when the left eye is given a drop and to the right when the right eye is being dropped and instantly wipe off the excessive fluid with a soft compress. Intervention will be given at several weeks of age and during a limited period.

**Table 1:** Administration schedule for eye drops according to ROP severity in affected eye/eyes

	**Number of drops per day**
ROP stage 1–2 without plus disease in zone I	1 drop every other day
ROP stage 2 without plus disease in posterior Zone II	1 drop every other day
ROP stage 3 without plus disease in zone II, with or without notch in zone 1	1 drop daily
Type 1 ROP* fulfilling treatment criteria	If anti-VEGF injection is planned, eye drops should be immediately withdrawn
ROP treatment and post treatment	Routines at the clinic

*Abbreviations:* *ROP* Retinopathy of prematurity, *VEGF* Vascular endothelial growth factor

*Any stage of ROP in zone I, with plus disease; ROP stage 3 in zone I, without plus disease or ROP stage 2 or 3 in zone II, with plus disease

The parents/guardians will record doses provided to the infant at home in the HOPE parent application. The total number of single doses used, along with compliance, will be estimated by collecting information about the number of doses returned and recorded in the HOPE application.

### Subject withdrawal

The parents/guardians can withdraw the infant from the study at any time. Infants may be withdrawn from the study by the attending neonatologist or the PI for safety reasons, such as adverse events (AE) (clinical events or laboratory values), major protocol deviations, or significant deteriorations in the patient’s condition that warrant discontinuation of the intervention. The final examination/sampling at study termination must then be performed at the time of the study discontinuation. Infants who withdraw from the study will not be replaced.

### Primary outcome of the intervention study

The primary objective is to evaluate if dexamethasone eye drops, compared to placebo, reduce the proportion of infants with severe ROP from progressing to severe Type 1 ROP needing treatment.

### Secondary outcomes of the intervention study and follow-up

The secondary objectives are to compare the following outcomes between the dexamethasone intervention and placebo:

- Time from detection of severe ROP to Type 1 ROP.
- Recurrences after laser/anti-VEGF treatment.
- Retinal morphology at 40 weeks postmenstrual age (PMA) and at 2 and 5.5 years of age.
- Change in intraocular pressure from before the intervention to 1–2 weeks after the intervention starts and at the end of the intervention.
- Visual acuity, refraction, orthoptic and ophthalmological status at 2 and 5.5 years of age.

### Exploratory outcomes of the intervention study and follow-up

Leftover blood from clinical blood sampling will be studied regarding selected biomarkers and their relation to ROP development and intervention effect.

### Safety outcomes in the intervention study

Clinically significant changes (abnormalities) from the physical examination of the infant or abnormal laboratory values will be recorded as an AE, starting from the time of randomization until the intervention phase termination visit. Ongoing AE at the intervention phase termination visit will be followed until the event is resolved or stable. Especially signs of increased ocular pressure (> 20 mmHg) or abnormal growth (> 1 standard deviation difference in weight, length and head circumference) after the start of intervention will be monitored as these are well-recognized side effects of high-dose steroid treatment, although this treatment is considered to be low dose. Other collected study-specific AEs will be pulmonary haemorrhage, pneumothorax, bronchopulmonary dysplasia (BPD), pulmonary hypertension, hypotension, circulatory instability, surgical patent ductus arteriosus (PDA), medical PDA, hypoglycemia, hyperglycemia, insulin treatment (glucose > 10 mmol/L at 2 independent time points), necrotising enterocolitis (NEC), perforated gut, ileus, enterostomy, sepsis, meningitis, intraventricular haemorrhage (IVH), and hydrocephalus. For each AE the following data will be recorded: AE diagnosis, AE start and end date, action taken regarding AE, AE outcome (resolved, ongoing, death, or lost to follow-up) and AE causality (not related, possibly related, related). Any severe AE (SAE), death or life-threatening event will be reported from the investigator at each site to the PI within 24 h of knowledge of the event. The local investigator and PI can stop the intervention temporarily or discontinue the infant from the study. If the infant is discontinued from the study, proper study termination procedures, including examinations and samplings, must be followed accordingly. The PI must report discontinuation due to an AE/SAE immediately to Thea Nordic.

### Study procedures and data collection at intervention phase visits

All data are collected and stored in agreement with good clinical practice guidelines and in accordance with the General Data Protection Regulation.

Table 2 presents the study schedule for visits and procedures in the “intervention phase” (main) study and the “extension” (follow-up) study. The intervention phase study termination visit is planned to occur approximately at PMA 40 weeks or two weeks after the eye drop intervention ends, regardless of regression of ROP or ROP treatment.

**Table 2:** DROPROP study schedule for study procedures and data collection

	**Intervention phase (Main) study**							**Extension study**	
**Procedures**	**ROP screening**	**Clinical visit**	**ROP screening/intervention**			**Clinical visit (40w PMA)**	**Termination visit**^**b**^	**Follow-up**	
	**Visit R0**	**Visit C1**	**Visit R1**	**Visit R2**	**Visit RX**^**a**^	**Visit C2**	**Visit T1**	**2 yrs** **Visit F1**	**5.5 yrs Visit F2**
**Data collection and study procedures**									
Informed consent	X								
Eligibility	X								
Medical history/characteristics of infant/mother	X								
ROP evaluations	X		X	X	X				
RetCam photography	X		X	X	X				
Randomization		X							
Dexamethasone/placebo intervention dispense/return		X	X	X	X	X	X		
ROP treatment with laser and/or anti-VEGF			X^c^	X^c^	X^c^				
Intraocular pressure	X^d^		X^d^	X^d^			X^d^		
MRI^e^ + Peapod						X^e^			
Physical examination		X				X		X	X
Weight, length, head circumference		X	X	X	X	X		X	X
Vital signs: SpO2, heart rate, blood pressure		X					X		
Blood samples		X^f^	X^f^	X^f^	X^f^	X^f^	X^f^		
Saliva cortisol		X	X	X	X		X	X	X
Urine sample		X				X	X		
Adverse events	X	X	X	X	X	X	X		
Visual outcome and refractive errors								X	X
Retinal morphology with OCT								X	X
Diagnoses and complications								X	X
Study termination		X	X	X	X	X	X	X	X

Study protocol version 4

Visit R0= Visit before start of intervention

Visit C1 = Clinical visit 1 (start of intervention)

Visit R1 = 1 week after start of intervention

Visit R2 = 2 weeks after start of intervention

Visit C2 = Clinical visit 2 (40 weeks PMA)

Visit T1 = 2 weeks after end of intervention

*Abbreviations*: *OCT* Coherence tomography, *PMA* Postmenstrual age, *ROP* Retinopathy of prematurity, *SpO2* Oxygen saturation, *VEGF* Vascular endothelial growth factor

^a^Visit RX = ROP Screening and intervention as many as required in order to be correctly followed-up regarding the monitoring and treatment of the ROP disease, procedures performed weekly

^b^Main study termination visit occurs two weeks after the last intervention or at earliest at 40 weeks PMA when maximum ROP should have occurred

^c^ROP treatment provided when required according to guidelines

^d^Intraocular pressure will be measured before intervention, 1 and 2 weeks after start of intervention and after end of intervention

^e^If performed in clinical routine, PeaPod in Gothenburg

^f^If clinical sample is taken left over blood is collected for analyses

The following data on infant and mother characteristics and medical history will be collected:

- Infant’s birth characteristics: GA at birth, sex, birth weight (grams), birth length (cm), birth head circumference (cm), single/twin/triplet, age at inclusion in the study.
- Infant’s medical history: lung morbidities like BPD, circulatory instability and PDA, gut problems like ileus and NEC, infections like sepsis, IVH and hydrocephalus. All surgical interventions.
- Mother’s age (years) and mother’s medical history: pregnancy complications, preeclampsia, infection requiring antibiotics, diabetes, hypertension or other disease requiring medication, parity, in vitro fertilization.

Intraocular pressure will be measured, and samples for salivary cortisol will be taken before intervention and thereafter according to the study protocol. Vital signs such as oxygen saturation (SpO2), heart rate and blood pressure will be measured before and after the intervention. At all clinical study visits, weight, length, and head circumference will be recorded. Body composition with PeaPod at 40 weeks PMA will be performed at sites where PeaPod is available. Brain Magnetic Resonance Imaging (MRI) will be performed at 40 weeks PMA according to clinical routine. Glucose in urine will be monitored after the initial start of intervention and at 40 weeks PMA. If intervention continues after 40 weeks PMA, a urine sample will be taken two weeks after the completion of intervention at the study termination visit. Leftover blood from clinical sampling will be analyzed for biomarkers involved in ROP-related processes such as angiogenesis, neurogenesis and inflammation (Gothenburg site).

### Extension study

In an extension part of the study, an ophthalmological follow-up at 2 and 5.5 years of age will evaluate the child’s best-corrected vision with an age-appropriate visual chart, refractive errors in cycloplegia, ocular motility, strabismus, nystagmus, and stereopsis. Retinal morphology with ocular coherence tomography (OCT) will be performed to evaluate the optic disc and maculae region. Neonatal and ophthalmological diagnoses and complications, as well as children’s growth parameters, will be collected at follow-up.

### Sample size

Natural history studies of ROP suggests that 50% of infants with ROP stage 2 in posterior zone II with haemorrhage or ROP stage 3 in zone II develop Type 1 ROP needing treatment [1]. We aim to reduce the number of infants progressing to severe ROP needing treatment with laser and/or anti-VEGF by 60%. Assuming 50% of infants need ROP treatment in the placebo group and 20% in the dexamethasone group, alpha 0.05, using a two-sided Fisher’s exact test, would require 45 infants to be included per study group (1:1 ratio), in total, 90 infants excluding dropouts, to achieve a power of 80%. Taking into account a 10% drop-out rate, five infants per study group and thus 100 infants in total will need to be included.

### Data monitoring

The data will be entered into a database, where internal review and programmed computer checks will be used to identify selected protocol violations and data errors. A statement will be obtained from each infant’s parents/guardians participating in the trial permitting the release of the infant’s medical records as necessary for monitoring or inspection by authorized personnel for the PI and Regulatory Authorities.

#### Case report forms

The PI will be responsible for the database containing the information collected in the study retrieved from the eCase Report Forms (eCRFs). The eCRFs will be developed using the HOPE platform from ADDI Medical (www.addimedical.se).

#### Data monitoring committee

A Data Monitoring Committee (DMC) will be appointed, including an independent group of experts in the area that are not the Study PI or part of the study’s steering committee, with one ophthalmologist, one neonatologist, and one epidemiologist, that will scrutinize the data prepared by a non-voting statistical programmer. Two planned closed DMC meetings will be held, the first when 35% of the infants have been followed until the main study ends, and the second when 70% of the infants have been followed until the main study ends. DMC will analyse data for efficacy (benefit) and safety (harm) at both time points. Ó’Brian-Flemming group sequential boundaries on the positive side will be applied for evaluation of potentially halting the trial because benefit has been shown, and a z-value exceeding 2.40 will be applied for evaluation of potentially halting the trial because harm has been shown. DMC may decide, given the descriptive data to not perform formal statistical interim tests if there is no sign of need for formal analyses for stopping for harm and stopping for efficacy. The DMC will not be responsible for stopping the study for futility. Given that the formal statistical interim tests are performed, the final *p*-values must be updated based on the number of interim tests performed. DMC may also, if necessary, schedule additional DMC meetings between planned closed meetings.

Information about study results will be strictly held within the closed DMC group. After a closed DMC meeting, the PI and the study’s steering committee will only receive a recommendation to stop or continue the study.

### Statistical analysis

All detailed statistical methods will be documented in a statistical analysis plan.

All analyses will be performed using either SAS software version 9.4 or later (SAS Institute Inc, Cary, NC, USA) or SPSS software version 26.0 or later (IBM Corp, Armonk, NY).

For tests between two groups, Fisher’s exact test will be used for dichotomous variables, Mantel–Haenszel Chi-square trend test for ordered categorical variables, Chi-square test for non-ordered categorical variables and Fisher’s nonparametric-permutation test for continuous variables. All efficacy evaluations will be adjusted for centre (strata in randomization) and gestational age (the most prominent risk factor). The difference between the two study arms with respect to binary outcomes such as the primary variable will be evaluated using logistic regression, for time-to-event data using Cox proportional hazards model, for number of recurrences per follow-up time using Poisson regression, for continuous outcomes using analysis of covariance (ANCOVA) or Fisher’s non-parametric permutation in case of not normally distributed data, and continuous outcomes repeatedly measured over time using mixed models for repeated measures.

All tests will be two-tailed. The primary analysis will be considered confirmed if *p* < 0.05.

### Ethical considerations

The protocol, informed consent form, and other written infant information have been submitted to the “Etikprövningsmyndigheten” (EPN, www.epn.se) in Sweden, (Ethical Dnr 2020–06028, Eudra-CT, 2020–004933 - 19 and CTIS, 2023–505318 - 97–00). The written unconditional approval was obtained prior to the study’s commencement and was forwarded to Thea Nordic, the manufacturer of the study medication, prior to shipment of study medication supplies to the centre. The study protocol has undergone independent peer-review to gain funding from a major external funding body.

## Discussion

This paper presents the DROPROP protocol: a prospective, randomized, multi-centre, double-blind trial designed to evaluate whether dexamethasone eye drops reduce the proportion of infants with severe ROP progressing to Type 1 ROP needing treatment compared to placebo. The findings of this study may impact the care of future preterm infants at risk of developing sight-threatening severe ROP worldwide.

As survival rates for preterm infants rise, the number of infants affected by sight-threatening ROP and needing ophthalmological expertise increases accordingly [2]. There is a concerning shortage of ophthalmologists available for ROP screening and treatment in many countries. We believe that dexamethasone eye drops could be a simple and low-cost treatment to reduce ophthalmological complications and lifelong visual impairment in these vulnerable new survivors. Research including preterm infants is challenging as they are particularly vulnerable and frequently experience critical illness and have uncertain prognoses [23]. Nevertheless, research is crucial, especially if it enhances our understanding or treatment of a medical condition [24]. Historically, clinical misadventures have occurred due to insufficient paediatric clinical research and off-label use of medications [25].

Research involving high-risk patients raises several ethical issues, particularly balancing potential risks and benefits. We acknowledge the safety concerns of topical dexamethasone intervention in our study. Intravenous or oral dexamethasone is commonly used in preterm care, i.e., to wean preterm infants from the ventilator and to prevent BPD. When used to prevent BPD, an initial high and then a tapering dose of 0.5 mg/kg per day of dexamethasone on the first day of life has been associated with an increased risk of cerebral palsy and impaired motor and cognitive development. However, treatment after one week of age and lower doses have not been shown to have the same adverse effects [21, 22]. The volume of a commercial eye drop is 50–75 µl, and the concentration of dexamethasone in Dexafree® eye drops is 1 mg/ml. Accordingly, we expose the infant to only 0.1–0.15 mg/day. Common ocular side effects of short-term topical dexamethasone are local discomfort during application and increased intraocular pressure, first noticed during the first two weeks of treatment (according to the product information of Dexafree®). We will, therefore, measure the intraocular pressure before and after one and two weeks of intervention and after ended intervention. Although systemic side effects are unlikely with the small doses of dexamethasone administered, we will closely monitor the infants’ weight and head circumference development as well as glucose levels in blood and urine during the intervention period and at 40 weeks PMA, a time point at which MRI of the brain will also be performed. We also address the risk of suppressed adrenal function and reduced endogenous cortisol production during intervention. Therefore, we collect saliva samples for cortisol measurement before and after intervention, weekly during intervention, and follow-ups at 2 and 5.5 years of age. We consider the use of local eye drops with a low dose of dexamethasone for a limited period (one to 12 weeks) to pose less risk to the infant than general anesthesia, laser and/or anti-VEGF treatment. The benefits of reducing potential risks associated with general anesthesia and destructive laser treatment, as well as exposing infants to anti-VEGF with unknown systemic effects, are substantial. Multiple studies have shown that infants treated for ROP have the poorest visual and cognitive outcomes [26, 27]. It is also well known that infants treated for ROP with laser and/or anti-VEGF have a higher risk for blindness than not developing ROP needing treatment [28, 29]. Both general anaesthesia and anti-VEGF-treatment are suspected to affect brain development [12, 30], and laser treatment is a destructive treatment that damages the peripheral retina [11].

In conclusion, we conduct this study to evaluate the effectiveness and safety of dexamethasone eye drops as a preventative therapy for preterm infants with severe ROP. The findings from our study aim to support clinical practice and national guidelines to provide the best care for this particularly vulnerable group of infants worldwide. We believe that our research has the potential to significantly benefit children by reducing the risk for visual impairments, thereby improving their quality of life.

We plan to publish the results of this study in peer-reviewed journals and present data at national and international conferences.

## Supplementary Information

Supplementary Material 1.

## Data Availability

No datasets were generated or analysed during the current study.

## Abbreviations

AE: Adverse Event
BPD: Bronchopulmonary Dysplasia
CRF: Case Report Form
DMC: Data Monitoring Committee
GA: Gestational Age
IVH: Intraventricular haemorrhage
NEC: Necrotizing enterocolitis
MRI: Magnetic Resonance Imaging
NICU: Neonatal Intensive Care Unit
PDA: Patent Ductus
PMA: Postmenstrual Age
ROP: Retinopathy of Prematurity
SAE: Serious Adverse Event
SpO2: Oxygen saturation

## References

[1] Shim JW, Jin HS, Bae CW. Changes in survival rate for very-low-birth-weight infants in korea: comparison with other countries. J Korean Med Sci. 2015;30 Suppl 1(Suppl 1):S25-34. 26566354 10.3346/jkms.2015.30.S1.S25 PMC4641060

[2] Lundgren P, Morsing E, Hård AL, Rakow A, Hellström-Westas L, Jacobson L, et al. National cohort of infants born before 24 gestational weeks showed increased survival rates but no improvement in neonatal morbidity. Acta Paediatr. 2022;111(8):1515–25. 35395120 10.1111/apa.16354 PMC9454067

[3] Blencowe H, Cousens S, Chou D, Oestergaard M, Say L, Moller AB, et al. Born too soon: the global epidemiology of 15 million preterm births. Reprod Health. 2013;10 Suppl 1(Suppl 1):S2. 24625129 10.1186/1742-4755-10-S1-S2 PMC3828585

[4] Holmström G, Hellström A, Gränse L, Saric M, Sunnqvist B, Wallin A, et al. New modifications of Swedish ROP guidelines based on 10-year data from the SWEDROP register. Br J Ophthalmol. 2020;104(7):943–9. 31676594 10.1136/bjophthalmol-2019-314874

[5] Gilbert C, Foster A. Childhood blindness in the context of VISION 2020–the right to sight. Bull World Health Organ. 2001;79(3):227–32. 11285667 PMC2566382

[6] Hellström A, Smith LE, Dammann O. Retinopathy of prematurity. Lancet. 2013;382(9902):1445–57. 23782686 10.1016/S0140-6736(13)60178-6 PMC4389630

[7] Holm M, Morken TS, Fichorova RN, VanderVeen DK, Allred EN, Dammann O, et al. Systemic Inflammation-Associated Proteins and Retinopathy of Prematurity in Infants Born Before the 28th Week of Gestation. Invest Ophthalmol Vis Sci. 2017;58(14):6419–28. 29260199 10.1167/iovs.17-21931 PMC5736326

[8] Pierce EA, Foley ED, Smith LE. Regulation of vascular endothelial growth factor by oxygen in a model of retinopathy of prematurity. Arch Ophthalmol. 1996;114(10):1219–28. 8859081 10.1001/archopht.1996.01100140419009

[9] Revised indications for the treatment of retinopathy of prematurity: results of the early treatment for retinopathy of prematurity randomized trial. Arch Ophthalmol. 2003;121(12):1684–94. 10.1001/archopht.121.12.1684 14662586

[10] The International Classification of Retinopathy of Prematurity revisited. Arch Ophthalmol. 2005;123(7):991–9. 16009843 10.1001/archopht.123.7.991

[11] Barnett JM, Hubbard GB. Complications of retinopathy of prematurity treatment. Curr Opin Ophthalmol. 2021;32(5):475–81. 34231532 10.1097/ICU.0000000000000783 PMC8373698

[12] Sankar MJ, Sankar J, Chandra P. Anti-vascular endothelial growth factor (VEGF) drugs for treatment of retinopathy of prematurity. Cochrane Database Syst Rev. 2018;1(1):Cd009734. 29308602 10.1002/14651858.CD009734.pub3 PMC6491066

[13] Bengani LC, Kobashi H, Ross AE, Zhai H, Salvador-Culla B, Tulsan R, et al. Steroid-eluting contact lenses for corneal and intraocular inflammation. Acta Biomater. 2020;116:149–61. 32814140 10.1016/j.actbio.2020.08.013 PMC8040324

[14] Iovino C, Mastropasqua R, Lupidi M, Bacherini D, Pellegrini M, Bernabei F, et al. Intravitreal Dexamethasone Implant as a Sustained Release Drug Delivery Device for the Treatment of Ocular Diseases: A Comprehensive Review of the Literature. Pharmaceutics. 2020;12(8):703. 10.3390/pharmaceutics12080703 PMC7466091 32722556

[15] Lee J, Dammann O. Perinatal infection, inflammation, and retinopathy of prematurity. Semin Fetal Neonatal Med. 2012;17(1):26–9. 21903492 10.1016/j.siny.2011.08.007 PMC3242877

[16] Rivera JC, Holm M, Austeng D, Morken TS, Zhou TE, Beaudry-Richard A, et al. Retinopathy of prematurity: inflammation, choroidal degeneration, and novel promising therapeutic strategies. J Neuroinflammation. 2017;14(1):165. 28830469 10.1186/s12974-017-0943-1 PMC5567917

[17] Rotschild T, Nandgaonkar BN, Yu K, Higgins RD. Dexamethasone reduces oxygen induced retinopathy in a mouse model. Pediatr Res. 1999;46(1):94–100. 10400141 10.1203/00006450-199907000-00016

[18] Yossuck P, Yan Y, Tadesse M, Higgins RD. Dexamethasone and critical effect of timing on retinopathy. Invest Ophthalmol Vis Sci. 2000;41(10):3095–9. 10967069

[19] Yagi H, Boeck M, Petrishka-Lozenska M, Lundgren P, Kasai T, Cagnone G, et al. Timed topical dexamethasone eye drops improve mitochondrial function to prevent severe retinopathy of prematurity. Angiogenesis. 2024;27(4):903–17. 39287727 10.1007/s10456-024-09948-2 PMC11564262

[20] Öhnell HM, Andreasson S, Gränse L. Dexamethasone Eye Drops for the Treatment of Retinopathy of Prematurity. Ophthalmol Retina. 2022;6(2):181–2. 34517147 10.1016/j.oret.2021.09.002

[21] Doyle LW, Cheong JL, Ehrenkranz RA, Halliday HL. Early (< 8 days) systemic postnatal corticosteroids for prevention of bronchopulmonary dysplasia in preterm infants. Cochrane Database Syst Rev. 2017;10(10):Cd001146. 29063585 10.1002/14651858.CD001146.pub5 PMC6485683

[22] Doyle LW, Cheong JL, Ehrenkranz RA, Halliday HL. Late (> 7 days) systemic postnatal corticosteroids for prevention of bronchopulmonary dysplasia in preterm infants. Cochrane Database Syst Rev. 2017;10(10):Cd001145. 29063594 10.1002/14651858.CD001145.pub4 PMC6485440

[23] Diekema DS. Ethical issues in research involving infants. Semin Perinatol. 2009;33(6):364–71. 19914520 10.1053/j.semperi.2009.07.003

[24] Modi N, Vohra J, Preston J, Elliott C, Van’t Hoff W, Coad J, et al. Guidance on clinical research involving infants, children and young people: an update for researchers and research ethics committees. Arch Dis Child. 2014;99(10):887–91. 24914095 10.1136/archdischild-2014-306444

[25] Fleischman AR. Ethical issues in neonatal research involving human subjects. Semin Perinatol. 2016;40(4):247–53. 26804381 10.1053/j.semperi.2015.12.014

[26] Morken TS, Dammann O, Skranes J, Austeng D. Retinopathy of prematurity, visual and neurodevelopmental outcome, and imaging of the central nervous system. Semin Perinatol. 2019;43(6):381–9. 31174874 10.1053/j.semperi.2019.05.012

[27] Diggikar S, Gurumoorthy P, Trif P, Mudura D, Nagesh NK, Galis R, et al. Retinopathy of prematurity and neurodevelopmental outcomes in preterm infants: A systematic review and meta-analysis. Front Pediatr. 2023;11:1055813. 37009271 10.3389/fped.2023.1055813 PMC10050340

[28] Lee BJ, Kim JH, Heo H, Yu YS. Delayed onset atypical vitreoretinal traction band formation after an intravitreal injection of bevacizumab in stage 3 retinopathy of prematurity. Eye (Lond). 2012;26(7):903–9 quiz 10. 22699977 10.1038/eye.2012.111 PMC3396159

[29] Emami S, Isaac M, Mireskandari K, Tehrani NN. Laser Treatment for Retinopathy of Prematurity: A Decade since ETROP. Ophthalmology. 2019;126(4):639–41. 30537485 10.1016/j.ophtha.2018.12.012

[30] Walsh BH, Paul RA, Inder TE, Shimony JS, Smyser CD, Rogers CE. Surgery requiring general anesthesia in preterm infants is associated with altered brain volumes at term equivalent age and neurodevelopmental impairment. Pediatr Res. 2021;89(5):1200–7. 32575110 10.1038/s41390-020-1030-3 PMC7755708

