# Supplementary material for: Evaluation of timed dexamethasone eye drops to prevent proliferative retinopathy of prematurity: a study protocol for a randomized intervention, multi-centre, double-blinded trial (DROPROP)
Source: BMC Pediatr. 2025 Apr 28;25:332. doi: 10.1186/s12887-025-05673-x (PMC12036246; doi:10.1186/s12887-025-05673-x)
Supplement: Supplementary file 1 — Supplementary Material 1. [file 12887_2025_5673_MOESM1_ESM.docx]

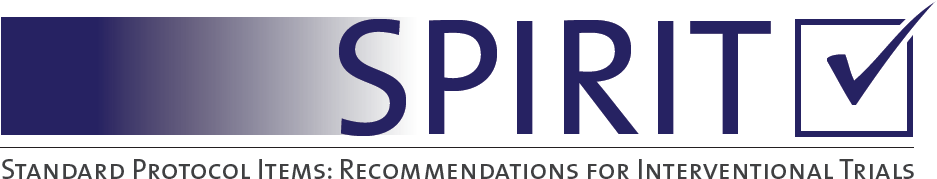


SPIRIT 2013 Checklist: Recommended items to address in a clinical trial protocol and

related documents*

| **Section/item** | **Item No** | **Description** |
| --- | --- | --- |
| **Administrative information** | | |
| Title | 1 | Descriptive title identifying the study design, population, interventions, and, if applicable, trial acronym  *Evaluation of timed dexamethasone eye drops to prevent proliferative retinopathy of prematurity: a study protocol for a randomized intervention, multi-centre, double-blinded trial (DROPROP)* |
| Trial registration | 2a | Trial identifier and registry name. If not yet registered, name of intended registry  *European Union Drug Regulating Authorities Clinical Trials Database, EudraCT#2020-004933-19 and Clinical Trials in the European Union, CTIS #2023-505318-97-00* |
|  | 2b | All items from the World Health Organization Trial Registration Data Set  *Not applicable* |
| Protocol version | 3 | Date and version identifier  *DROPROP protocol October 2022, version 4* |
| Funding | 4 | Sources and types of financial, material, and other support  *This study was supported by grants provided by the Swedish Research Council (2015-00810, 2016-01131, and 2022-01562), the Swedish state under the agreement between the Swedish government and the county councils - the ALF-agreement (ALFGBG-812951, ALFGBG-971188, ALF 2022-YF0008), The Wallenberg Clinical Scholars (KAW 2018.0310), the SciLifeLab & Wallenberg Data Driven Life Science Program (KAW 2020.0239), NIH EY017017, EY030904-01, BCH IDDRC (1U54HD090255) and Massachusetts Lions Eye Foundation.* |
| Roles and responsibilities | 5a | Names, affiliations, and roles of protocol contributors  *Ann Hellström1,2*, Mariya Petrishka-Lozenska1,2, Aldina Pivodic1,2, Anders K Nilsson1, Ulrika Sjöbom1, Ingrid Hansen Pupp3, David Ley3, Lotta Gränse4, Hanna Maria Öhnell4, Gunnar Jakobsson5, Karin Sävman6.7, Lois E.H Smith8, Pia Lundgren1,2* and the DROPROP collaboration group9*  *1 The Sahlgrenska Centre for Pediatric Ophthalmology Research, Department of Clinical Neuroscience, Institute of Neuroscience and Physiology, Sahlgrenska Academy, University of*  *Gothenburg, Gothenburg, Sweden.*  *2 Department of Ophthalmology, Sahlgrenska University Hospital, Region Västra Götaland, Gothenburg, Sweden*  *3 Department of Clinical Sciences Lund, Pediatrics, Lund University, Skåne University Hospital, Lund, Sweden*  *4 Department of Clinical Sciences Lund, Ophthalmology, Lund University, Skåne University Hospital, Lund, Sweden.*  *5 Department of Ophthalmology, Sahlgrenska University Hospital, Region Västra Götaland, Mölndal, Sweden*  *6 Department of Pediatrics, Institute of Clinical Sciences, Sahlgrenska Academy, University of Gothenburg, Gothenburg, Sweden.*  *7 Region Västra Götaland, Dept of Neonatology, The Queen Silvia Children's Hospital, Sahlgrenska University Hospital, Gothenburg, Sweden.*  *8 The Department of Ophthalmology, Boston Children's Hospital, Harvard Medical School, Boston, MA., USA.*  *9DROPROP collaboration group: Magnus Domellöf10, Kristina Teär Fahnehjelm11,12, Uwe Ewald13, Eva Albinsson13, Erik Normann13, Afsaneh Alibakhshi14, Nikica Tomašić15,16, Fredrik Ingemansson17,18, Pierfrancesco Mirabelli19,20, Despina Tsamadou11,21, Jenny Wallander22, Evangelos Tsigkoulis19, Stefan Löfgren23, Sofie Eriksson24, Karin Hochhard Sandgren25, Ylva Friberg-Riad2, Liv Vallin26*  *10 Department of Clinical Sciences, Pediatrics, Umeå University, Umeå, Sweden.*  *11Department of Clinical Neuroscience, Karolinska Institutet, Stockholm, Sweden.*  *12Department of Paediatric Ophthalmology, Strabismus and Electrophysiology, St. Erik Eye Hospital, Stockholm, Sweden.*  *13Department of Women’s and Children’s Health, Uppsala University, Uppsala, Sweden.*  *14Department of Ophthalmology, Southern Älvsborg Hospital, Borås, Sweden.*  *15Department of Neonatology, Karolinska University Hospital, Stockholm, Sweden.*  *16Division of Pediatric Endocrinology and Metabolism, Karolinska University Hospital, Stockholm, Sweden.*  *17Department of Pediatrics, Region Jönköping County, Jönköping, Sweden.*  *18Department of Clinical and Experimental Medicine, Linköping University, Linköping, Sweden.*  *19Department of Ophthalmology, Linköping University, Linköping, Sweden.*  *20Department of Biomedical and Clinical Sciences, Linköping University, Linköping, Sweden.*  *21Department of Ophthalmology, Örebro University Hospital, Örebro, Sweden.*  *22Department of Ophthalmology, Region Jönköping County, Jönköping, Sweden.*  *23St. Erik Eye Hospital, Karolinska Institutet, Stockholm, Sweden.*  *24Department of Ophthalmology, Region Värmland, Karlstad, Sweden.*  *25Ophthalmology, Department of Clinical Sciences, Umeå University, Umeå, Sweden.*  *26Department of Ophthalmology. Östersund Hospital, Östersund, Sweden.* |
|  | 5b | Name and contact information for the trial sponsor  *Professor Ann Hellström is the primary sponsor and principal investigator (PI) in this study.* [*ann.hellstrom@medfak.gu.se*](mailto:ann.hellstrom@medfak.gu.se) |
|  | 5c | Role of study sponsor and funders, if any, in study design; collection, management, analysis, and interpretation of data; writing of the report; and the decision to submit the report for publication, including whether they will have ultimate authority over any of these activities  *A.H. is the principal investigator of this study. A.H., A.P., I. H P., D.L., L.G., K.S., and L.E.H.S, conceived and designed the initial draft of the study protocol with contributions from P.L., M.P-L., A.K.N., H M.Ö., G.J. and U.S. All authors were involved in the preconsensus process of the protocol. A.P. is the study statistician. P.L. wrote the initial draft of this manuscript based on the study protocol. All authors revised the manuscript, made important contributions and approved the final version. All authors revised the manuscript, made important contributions and approved the final version.* |
|  | 5d | Composition, roles, and responsibilities of the coordinating centre, steering committee, endpoint adjudication committee, data management team, and other individuals or groups overseeing the trial, if applicable (see Item 21a for data monitoring committee)  *The steering group is composed of three experienced pediatric ophthalmologists.* *A Data Monitoring Committee (DMC) will be appointed, including an independent group of experts in the area that are not the Study PI or part of the study’s steering committee, with one ophthalmologist, one neonatologist, and one epidemiologist, that will scrutinize the data prepared by a non-voting statistical programmer.* |
| **Introduction** |  |  |
| Background and rationale | 6a | Description of research question and justification for undertaking the trial, including summary of relevant studies (published and unpublished) examining benefits and harms for each intervention  *Studies on the efficacy and safety of dexamethasone eye drops to prevent severe ROP needing treatment in preterm infants are warranted. We designed a prospective, randomized, double-blinded, controlled, multi-centre intervention study to include preterm infants with severe ROP not fulfilling treatment criteria, i.e. infants with Type 2 ROP and infants with stage 2 in posterior zone II. We aim to reduce the number of infants with severe ROP needing treatment by 60% with the dexamethasone intervention compared to placebo.*  *Pages 6-9 in the manuscript.* |
|  | 6b | Explanation for choice of comparators  *Study patients will be randomized to receive eyedrops with either dexamethasone 1 mg/ml (Dexafree®) in single-dose containers supplied by Thea Nordic or placebo, saline (Drop-it®) also in single-dose containers. Saline (Drop-it®) is a sterile, buffered saline without preservatives, with the same sodium chloride content as the tear film.* |
| Objectives | 7 | Specific objectives or hypotheses  *The primary objective is to evaluate if dexamethasone eye drops, compared to placebo, reduce the proportion of infants with severe ROP from progressing to severe Type 1 ROP needing treatment.* |
| Trial design | 8 | Description of trial design including type of trial (eg, parallel group, crossover, factorial, single group), allocation ratio, and framework (eg, superiority, equivalence, noninferiority, exploratory)  *The study is a national multi-centre, randomized, double-blinded, controlled prospective study.* |

| **Methods: Participants, interventions, and outcomes** | | |
| --- | --- | --- |
| Study setting | 9 | Description of study settings (eg, community clinic, academic hospital) and list of countries where data will be collected. Reference to where list of study sites can be obtained  *The following centres in Sweden participated in the study: Sahlgrenska University Hospital (Gothenburg), Karolinska University Hospital (Stockholm), the university hospitals in Umeå, Uppsala, Örebro and* *Linköping, Södra Älvsborgs Hospital, Skaraborgs hospital (Skövde), Ryhov hospital (Jönköping), and the hospitals in Karlstad, Växjö, Gävle, Östersund, Västerås and Eskilstuna.* |
| Eligibility criteria | 10 | Inclusion and exclusion criteria for participants. If applicable, eligibility criteria for study centres and individuals who will perform the interventions (eg, surgeons, psychotherapists)  *Infants are eligible if they fulfil the following criteria:*  *• Infants born with a gestational age (GA) <30 weeks.*  *• ROP stage 1 or stage 2 without plus disease in zone I.*  *• ROP stage 2 or stage 3 in posterior zone II without plus disease, with or without notch in zone I.*  *• ROP severity must be documented using wide-field digital imaging system photography (RetCam), and eligibility must be confirmed by at least two of three experienced paediatric ophthalmologists in the steering group.*  *• Signed informed consent by the parents/guardians (oral and written information).*  *The exclusion criteria are:*  *• Ongoing ocular infection.*  *• If the neonatologist or ophthalmologist considers the infant unsuitable for the study.* |
| Interventions | 11a | Interventions for each group with sufficient detail to allow replication, including how and when they will be administered  *Based on the severity of ROP, the eye drops are administered at a dosage of one drop every other day to one drop daily in affected eyes, Table 1. The maximum intervention duration is 12 weeks.* |
|  | 11b | Criteria for discontinuing or modifying allocated interventions for a given trial participant (eg, drug dose change in response to harms, participant request, or improving/worsening disease)  *The parents/guardians can withdraw the infant from the study at any time. Infants may be withdrawn from the study by the attending neonatologist or the PI for safety reasons, such as adverse events (AE) (clinical events or laboratory values), major protocol deviations, or significant deteriorations in the patient’s condition that warrant discontinuation of the intervention. The final examination/sampling at study termination must then be performed at the time of the study discontinuation. Infants who withdraw from the study will not be replaced.* |
|  | 11c | Strategies to improve adherence to intervention protocols, and any procedures for monitoring adherence (eg, drug tablet return, laboratory tests)  *The parents/guardians will record doses provided to the infant at home in the HOPE parent application. The total number of single doses used, along with compliance, will be estimated by collecting information about the number of doses returned and recorded in the HOPE application.* |
|  | 11d | Relevant concomitant care and interventions that are permitted or prohibited during the trial  *Not applicable* |
| Outcomes | 12 | Primary, secondary, and other outcomes, including the specific measurement variable (eg, systolic blood pressure), analysis metric (eg, change from baseline, final value, time to event), method of aggregation (eg, median, proportion), and time point for each outcome. Explanation of the clinical relevance of chosen efficacy and harm outcomes is strongly recommendedPrimary outcome of the intervention study  *The primary objective is to evaluate if dexamethasone eye drops, compared to placebo, reduce the proportion of infants with severe ROP from progressing to severe Type 1 ROP needing treatment.*  *Secondary outcomes of the intervention study and follow-up*  *The secondary objectives are to compare the following outcomes between the dexamethasone intervention and placebo:*  *• Time from detection of severe ROP to Type 1 ROP.*  *• Recurrences after laser/anti-VEGF treatment.*  *• Retinal morphology at 40 weeks postmenstrual age (PMA) and at 2 and 5.5 years of age.*  *• Change in intraocular pressure from before the intervention to 1-2 weeks after the intervention starts and at the end of the intervention.*  *• Visual acuity, refraction and orthoptic status at 2 and 5.5 years of age.* |
| Participant timeline | 13 | Time schedule of enrolment, interventions (including any run-ins and washouts), assessments, and visits for participants. A schematic diagram is highly recommended (see Figure)  *Figure 1 in the manuscript* |
| Sample size | 14 | Estimated number of participants needed to achieve study objectives and how it was determined, including clinical and statistical assumptions supporting any sample size calculations  *Natural history studies of ROP suggests that 50% of infants with ROP stage 2 in posterior zone II with haemorrhage or ROP stage 3 in zone II develop Type 1 ROP needing treatment (1). We aim to reduce the number of infants progressing to severe ROP needing treatment with laser and/or anti-VEGF by 60%. Assuming 50% of infants need ROP treatment in the placebo group and 20% in the dexamethasone group, alpha 0.05, using a two-sided Fisher’s exact test, would require 45 infants to be included per study group (1:1 ratio), in total, 90 infants excluding dropouts, to achieve a power of 80%. Taking into account a 10% drop-out rate, five infants per study group and thus 100 infants in total will need to be included*. |
| Recruitment | 15 | Strategies for achieving adequate participant enrolment to reach target sample size  *Infants evaluated for eligibility for enrollment in the study will be identified at routine ROP screening by the local ophthalmologist.* |
| **Methods: Assignment of interventions (for controlled trials)** | | |
| Allocation: |  |  |
| Sequence generation | 16a | Method of generating the allocation sequence (eg, computer- generated random numbers), and list of any factors for stratification. To reduce predictability of a random sequence, details of any planned restriction (eg, blocking) should be provided in a separate document that is unavailable to those who enrol participants or assign  Interventions  *Randomization will be performed stratified by center using an electronic application. The block size was not revealed to the study personnel.* |

| Allocation concealment mechanism | 16b | Mechanism of implementing the allocation sequence (eg, central telephone; sequentially numbered, opaque, sealed envelopes), describing any steps to conceal the sequence until interventions are assigned  *Randomization will be performed stratified by centre by a study nurse using an electronic application (HOPE).* |
| --- | --- | --- |
| Implementation | 16c | Who will generate the allocation sequence, who will enrol participants, and who will assign participants to interventions  *Images will be evaluated, and eligibility for inclusion must be confirmed by at least two of three ophthalmologists in the steering group. Randomization will be performed stratified by centre by a study nurse using an electronic application (HOPE). Infants eligible for the study will be randomized in strictly sequential order at each center according to the study’s randomization list. They will be randomized to receive eye drops with either Dexamethasone 1 mg/ml (Dexafree®) or physiologic saline provided in similar single-dose containers. Treatment will be of single-blind nature, blinded for the ophthalmologist treating the infants. Each randomization number will have a sealed envelope available at the center, stored safely in a locker where the randomized treatment is listed in case of safety issues and need for unblinding among the study personnel.* |
| Blinding (masking) | 17a | Who will be blinded after assignment to interventions (eg, trial participants, care providers, outcome assessors, data analysts), and how  *All investigators, ophthalmologists, staff, and patients/guardians, except pharmacists and the nurse performing the randomizations, are blind to the participants’ eye drop assignments.* |
|  | 17b | If blinded, circumstances under which unblinding is permissible, and procedure for revealing a participant’s allocated intervention during the trial  *In case of problems regarding the study eye drops, a randomization list is safely stored in a locker at the NICU and available 24 hours/day in case of emergency and safety issues.* |
| **Methods: Data collection, management, and analysis** | | |
| Data collection methods | 18a | Plans for assessment and collection of outcome, baseline, and other trial data, including any related processes to promote data quality (eg, duplicate measurements, training of assessors) and a description of study instruments (eg, questionnaires, laboratory tests) along with their reliability and validity, if known. Reference to where data collection forms can be found, if not in the protocol  *Table 2 in the manuscript.* |
|  | 18b | Plans to promote participant retention and complete follow-up, including list of any outcome data to be collected for participants who discontinue or deviate from intervention protocols  *Parents/guardians will be given oral and written information about the study via the HOPE Parent Application (Addi Medical).*  *The parents/guardians can withdraw the infant from the study at any time. Infants may be withdrawn from the study by the attending neonatologist or the PI for safety reasons, such as adverse events (AE) (clinical events or laboratory values), major protocol deviations, or significant deteriorations in the patient’s condition that warrant discontinuation of the intervention. The final examination/sampling at study termination must then be performed at the time of the study discontinuation. Infants who withdraw from the study will not be replaced.* |
| Data management | 19 | Plans for data entry, coding, security, and storage, including any related processes to promote data quality (eg, double data entry; range checks for data values). Reference to where details of data management procedures can be found, if not in the protocol  *The data will be entered into a database, where internal review and programmed computer checks will be used to identify selected protocol violations and data errors. A statement will be obtained from each infant’s parents/guardians participating in the trial permitting the release of the infant’s medical records as necessary for monitoring or inspection by authorized personnel for the PI and Regulatory Authorities.* |
| Statistical methods | 20a | Statistical methods for analysing primary and secondary outcomes. Reference to where other details of the statistical analysis plan can be found, if not in the protocol  *Pages 20-21 in the manuscript* |
|  | 20b | Methods for any additional analyses (eg, subgroup and adjusted analyses)  *Pages 20-21 in the manuscript.* |
|  | 20c | Definition of analysis population relating to protocol non-adherence (eg, as randomised analysis), and any statistical methods to handle missing data (eg, multiple imputation)  *The main study analyses will be performed on the intention-to-treat population, including all eligible, randomized infants. A per protocol analysis will include all eligible, randomized infants without any significant protocol violations. The safety analyses will include all infants that have received at least one dose of either product or placebo.* |
| **Methods: Monitoring** | | |
| Data monitoring | 21a | Composition of data monitoring committee (DMC); summary of its role and reporting structure; statement of whether it is independent from the sponsor and competing interests; and reference to where further details about its charter can be found, if not in the protocol.  Alternatively, an explanation of why a DMC is not needed  *A Data Monitoring Committee (DMC) will be appointed, including an independent group of experts in the area that are not the Study PI or part of the study’s steering committee, with one ophthalmologist, one neonatologist, and one epidemiologist, that will scrutinize the data prepared by a non-voting statistical programmer.* |

|  | 21b | Description of any interim analyses and stopping guidelines, including who will have access to these interim results and make the final decision to terminate the trial  *Two planned closed DMC meetings will be held, the first when 35% of the infants have been followed until the main study ends, and the second when 70% of the infants have been followed until the main study ends. DMC will analyse data for efficacy (benefit) and safety (harm) at both time points. Ó’Brian-Flemming group sequential boundaries on the positive side will be applied for evaluation of potentially halting the trial because benefit has been shown, and a z-value exceeding 2.40 will be applied for evaluation of potentially halting the trial because harm has been shown. DMC may decide, given the descriptive data to not perform formal statistical interim tests if there is no sign of need for formal analyses for stopping for harm and stopping for efficacy. The DMC will not be responsible for stopping the study for futility. Given that the formal statistical interim tests are performed, the final p-values must be updated based on the number of interim tests performed. DMC may also, if necessary, schedule additional DMC meetings between planned closed meetings.*  *Information about study results will be strictly held within the closed DMC group. After a closed DMC meeting, the PI and the study’s steering committee will only receive a recommendation to stop or continue the study.* |
| --- | --- | --- |
| Harms | 22 | Plans for collecting, assessing, reporting, and managing solicited and spontaneously reported adverse events and other unintended effects of trial interventions or trial conduct.  *Clinically significant changes (abnormalities) from the physical examination of the infant or abnormal laboratory values will be recorded as an AE, starting from the time of randomization until the intervention phase termination visit. .... Any severe AE (SAE), death or life-threatening event will be reported from the investigator at each site to the PI within 24 hours of knowledge of the event. The local investigator and PI can stop the intervention temporarily or discontinue the infant from the study. If the infant is discontinued from the study, proper study termination procedures, including examinations and samplings, must be followed accordingly. The PI must report discontinuation due to an AE/SAE immediately to Thea Nordic*. |
| Auditing | 23 | Frequency and procedures for auditing trial conduct, if any, and whether the process will be independent from investigators and the sponsor.  *DMC will be responsible for reviewing protocol adherence and data quality.* |
| **Ethics and dissemination** | | |
| Research ethics approval | 24 | Plans for seeking research ethics committee/institutional review board (REC/IRB) approval  *Ethical approval is finalized.* |
| Protocol amendments | 25 | Plans for communicating important protocol modifications (eg, changes to eligibility criteria, outcomes, analyses) to relevant parties (eg, investigators, REC/IRBs, trial participants, trial registries, journals, regulators)  *Communication about protocol modifications will to regulators, trials participants, research personnel and investigators will be performed when needed.* |
| Consent or assent | 26a | Who will obtain informed consent or assent from potential trial participants or authorised surrogates, and how (see Item 32)  *Parents/guardians will be given oral and written information about the study via the HOPE Parent Application (Addi Medical) and asked to participate if the infant is eligible. The signed informed consent will be collected before any study-specific data is reported and the infant is randomized.* |
|  | 26b | Additional consent provisions for collection and use of participant data and biological specimens in ancillary studies, if applicable  *Not applicable* |
| Confidentiality | 27 | How personal information about potential and enrolled participants will be collected, shared, and maintained in order to protect confidentiality before, during, and after the trial.  *The data will be entered into a database, where internal review and programmed computer checks will be used to identify selected protocol violations and data errors. A statement will be obtained from each infant’s parents/guardians participating in the trial permitting the release of the infant’s medical records as necessary for monitoring or* *inspection by authorized personnel for the PI and Regulatory Authorities.*  *The PI will be responsible for the database containing the information collected in the study retrieved from the eCase Report Forms (eCRFs). The eCRFs will be developed using the HOPE platform from ADDI Medical (www.addimedical.se).* |
| Declaration of interests | 28 | Financial and other competing interests for principal investigators for the overall trial and each study site  *The investigational products, Dexafree® and Drop-it® were donated by Théa Nordic AB at no cost. A.H. is the sister of Nina Sellberg, who is the chief executive officer of Addi Medical AB; A.H. has no financial interests in Addi Medical AB. All authors declare that they have no competing interests.* |
| Access to data | 29 | Statement of who will have access to the final trial dataset, and disclosure of contractual agreements that limit such access for investigators.  *The PI will be responsible for the database containing the information collected in the study retrieved from the eCase Report Forms (eCRFs). The eCRFs will be developed using the HOPE platform from ADDI Medical (www.addimedical.se).* |
| Ancillary and post-trial care | 30 | Provisions, if any, for ancillary and post-trial care, and for compensation to those who suffer harm from trial participation  *Not applicable.* |
| Dissemination policy | 31a | Plans for investigators and sponsor to communicate trial results to participants, healthcare professionals, the public, and other relevant groups (eg, via publication, reporting in results databases, or other data sharing arrangements), including any publication restrictions  *We plan to publish the results of this study in peer-reviewed journals and present data at national and international conferences.* |
|  | 31b | Authorship eligibility guidelines and any intended use of professional writers  *A.H. is the principal investigator of this study. A.H., A.P., I. H P., D.L., L.G., K.S., and L.E.H.S, conceived and designed the initial draft of the study protocol with contributions from P.L., M.P-L., A.K.N., H M.Ö., G.J. and U.S. All authors were involved in the preconsensus process of the protocol. A.P. is the study statistician. P.L. wrote the initial draft of this manuscript based on the study protocol. All authors revised the manuscript, made important contributions and approved the final version. All authors revised the manuscript, made important contributions and approved the final version.* |
|  | 31c | Plans, if any, for granting public access to the full protocol, participant- level dataset, and statistical code  *Data sharing is not applicable to this article*. |

| **Appendices** |  |  |
| --- | --- | --- |
| Informed consent materials | 32 | Model consent form and other related documentation given to participants and authorised surrogates  *Model consent form and additional information about independent peer-review has been uploaded as supplements.* |
| Biological specimens | 33 | Plans for collection, laboratory evaluation, and storage of biological specimens for genetic or molecular analysis in the current trial and for future use in ancillary studies, if applicable  *Not applicable* |

*It is strongly recommended that this checklist be read in conjunction with the SPIRIT 2013 Explanation & Elaboration for important clarification on the items. Amendments to the protocol should be tracked and dated. The SPIRIT checklist is copyrighted by the SPIRIT Group under the Creative Commons “[Attribution-NonCommercial-NoDerivs 3.0 Unported](http://www.creativecommons.org/licenses/by-nc-nd/3.0/)” license.
